# Food addiction: a valid concept?

**DOI:** 10.1038/s41386-018-0203-9

**Published:** 2018-09-06

**Authors:** Paul C. Fletcher, Paul J. Kenny

**Affiliations:** 1 Department of Psychiatry, University of Cambridge, Cambridge, CB2 8AH UK; 2 Cambridgeshire and Peterborough NHS Foundation Trust, Cambrdge, CB21 5EF, UK; 3 Department of Neuroscience, Icahn School of Medicine at Mount Sinai, New York, NY 10029 USA

## Abstract

Can food be addictive? What does it mean to be a food addict? Do common underlying neurobiological mechanisms contribute to drug and food addiction? These vexing questions have been the subject of considerable interest and debate in recent years, driven in large part by the major health concerns associated with dramatically increasing body weights and rates of obesity in the United States, Europe, and other regions with developed economies. No clear consensus has yet emerged on the validity of the concept of food addiction and whether some individuals who struggle to control their food intake can be considered food addicts. Some, including Fletcher, have argued that the concept of food addiction is unsupported, as many of the defining features of drug addiction are not seen in the context of feeding behaviors. Others, Kenny included, have argued that food and drug addiction share similar features that may reflect common underlying neural mechanisms. Here, Fletcher and Kenny argue the merits of these opposing positions on the concept of food addiction.

## Arguments against food addiction (PCF)

“I am asked by the learned doctor the cause and reason why opium causes sleep. To which I reply: because it has a dormitive property [virtus dormitiva], whose nature is to lull the senses to sleep.”

—Moliere, La malade imaginaire

Applying diagnoses to make sense of collections of symptoms and behaviors has advantages in sharpening communication and clarifying thought. It may carry benefits for the patient, too, offering a model that seems to make sense of what is otherwise baffling. But the hope, if we are simply to avoid the circularity alluded to in the quote above or, as Jaspers put, it “pseudo-insight through terminology” [1], is to go beyond simple naming and to reach an understanding of processes and mechanisms. The application of the term “food addiction” in humans is based on a set of features, held to resemble substance addictions. It carries the claim that this resemblance occurs because certain foods have effects on the brain comparable to those of addictive drugs. I suggest that there are problems with both of these claims. The first is questionable as the central features of substance addiction do not plausibly translate to food and consumption. The second because the assertion that foods have pharmacological effects on the brain demands strong and convincing evidence, which has not been found.

Food addiction, as an explanation for the often-distressing cravings, loss of control, and overconsumption experienced by many, particularly in relation to highly palatable foods, has been with us for many years [2] and, having more latterly becomes a focus for direct scientific study, the model has sought support in two broad sets of work. First, clinico-behavioral work has produced a descriptive framework based on the asserted resemblance between overconsumption of food and substance use. This has led to the development of a widely used scale [3], which measures the characteristics thought to be common to substance and, putatively, food addiction (i.e., craving, loss of control, excessive consumption, tolerance, withdrawal, and distress/dysfunction). Scores on this scale have subsequently been used as the basis for interpreting variance in functional neuroimaging measures, with such correlations being cited as evidence for the neurobiological reality of food addiction. Second, insights into the neurobiology of substance use and addiction have generated hypotheses in rodent and human work, particularly focusing on reward processing and the mesolimbic dopamine system. Such studies have established that particular regimens of palatable food availability may produce addiction-like brain changes, as well as binge-like eating and withdrawal symptoms. It is important to acknowledge that there are some differing positions held by supporters of the food addiction model, but the prevailing view seems reasonably characterized as follows: with exceptions [4], food addiction is considered as separable from obesity. While one study shows that 88% meeting criteria for food addiction are obese [5], it is important to note that food addiction is defined by behavioral patterns and experiences relating to eating rather than by weight status. Moreover, it is considered to be separate from those established clinical conditions with which it markedly overlaps, notably eating disorders marked by binge eating [2]. It is identified by some self-reported combination of the above features and held to resemble a substance addiction rather than a behavioral addiction (though see ref. [6]), such as gambling disorder [7]. Critically, the suggestion goes, it is associated with corresponding changes to the mesolimbic dopamine system, changes that underpin the transition from reward-driven to impulsive and compulsive eating.

There are several problems with this model. First, the addictive substance remains undiscovered—a problem that cannot be considered trivial. The model rests on a central assertion that either some category of foods, or some specific nutrients, exert a direct effect on the brain, enacting changes that ultimately hijack reward-related behaviors. Some have argued that sugar is the culprit though, as a whole, the evidence for sugar addiction remains deeply unconvincing [8]. Alternatively, others maintain that the refined and excessively palatable combinations of sugars and fats in Western diets increase addiction liability. But, as of now, no addictive substance has been identified. Second, there are crucial questions relating to how food addiction fits into the overall schema of problematic eating. Though initially applied to understanding obesity as a whole, the current view is that food addiction represents a specific construct that is strongly argued to be distinct even from the eating disorder that perhaps it most resembles: binge-eating disorder [2]. This clear distinction is challenged, however, by studies showing marked overlap between positive scores for food addiction (as measured on a dedicated scale—the Yale Food Addiction Scale (YFAS; see below)) and binge eating in the context of bulimia nervosa [9–12]. The overlap too between food addiction and binge-eating disorder is striking [13]. One conclusion from this might be that food addiction is not distinct from the symptom of binge eating, perhaps with food addiction (and its putative underlying neurobiological imbalance) providing a mechanistic explanation for the latter. However, it has been argued that these are distinct entities and should not be conflated [2]. There is a pressing need, therefore, to assess the discriminant validity of the scale. It is crucial, too, that we consider the validity and reliability of other characteristics held to be a feature of food addiction, most notably tolerance and withdrawal. For example, eating increased amounts of food and obtaining less pleasure from eating is taken as evidence of tolerance, while anxiety, dysphoria, and unspecified “physical symptoms” during abstinence from certain foods are viewed as withdrawal symptoms. It is inevitable that brief, non-interactive clinical scales will be imprecise. It is important, though, that scales are not taken to validate the phenomenon that they are seeking to measure [14]. Further considerations of the problems in applying the diagnosis of food addiction clinically have been discussed elsewhere [14, 15].

Going beyond the phenotype, a critical aspect of the model, and one that moves it beyond a simple phenomenological description of a cluster of behaviors and symptoms, is the hypothesized set of neurobiological changes that underlie food addiction. The third concern therefore is especially important: there remains no convincing demonstration in humans that such neurobiological changes do indeed underlie these food addiction behaviors. We should see specifically altered structural and functional patterns in key nodes of the mesolimbic dopamine system in groups of individuals who exhibit food addiction behaviors. An early study suggested that this was indeed the case with reduced striatal dopamine receptor density in severely obese people [16]. But this finding, which is frequently cited in unqualified support for the food addiction model of obesity, has not been replicated and has been succeeded by several studies, which do not find such differences; e.g., refs. [17–19]. Reviewing the literature some 5 years ago, I and colleagues [20, 21] suggested that the existing functional magnetic resonance imaging and positron-emission tomography studies should not be used as a basis for making claims about food addiction. Reviewing the field now, there is no more support for food addiction from human neuroimaging literature than there was then. Indeed, there is less.

Fourth, there are several studies showing that controlled manipulation of food composition and availability provokes addiction-like patterns in rats. These studies convincingly demonstrate that experimentally constrained, intermittent availability of high-sugar, high-fat, or high-sugar plus high-fat diets provokes both compulsive patterns of eating in rats and a number of sequelae suggestive of addiction: notably, reduced dopamine receptor function, increased self-stimulation thresholds, and, in the case of sugar, anxious behaviors that may be relatable to withdrawal symptoms. Such studies constitute the most compelling evidence that highly palatable foods administered intermittently can produce changes in brain and behavior that replicate substance addiction. But, outstanding questions remain about whether and how these carefully controlled regimens translate to humans who, by and large, inhabit a very different food environment, one that is often characterized by constant and plentiful availability rather than constraint. To be clear, I do not deny the importance and value of these rodent models in developing our understanding of perturbations in food behaviors, but their emergence challenges us to translate them to humans, a challenge that has not been met. And it should be noted too that they raise important questions (see ref. [8] for fuller discussion): for example, it has been argued that it is the uncertainty of availability, rather than the emergence of addiction, that generates compulsive eating [22]. Moreover, while rats with intermittent access to a high-calorie food develop habit-like responding and corresponding activation in dorsolateral striatum, those with continued access to the same foods do not show this pattern [23].

When first expressing misgivings about the ready acceptance of food addiction as an explanation for obesity [20], our paper generated a brief correspondence [24] in which we all concluded with the cliched but invariably true suggestion that “more research is needed”. Reviewing the current literature, I suggest that little clarity has emerged: the case has not been further made that there is a convincing resemblance between problem food behaviors and substance addiction. Attempts to pinpoint the neurobiology of food addiction in humans have generated no consistent or replicated finding, overall failing to support the model and, in many areas, seeming quite strongly to contradict it. Furthermore, the most promising area of research, the modeling of addictive-like eating in rodents, has not progressed appreciably and, importantly, has not been followed-up in humans in a systematic way. And yet, there is a continued willingness to diagnose, measure, and apply food addiction as a validated concept in research.

The strong narrative around food addiction is, of course, understandable. A person prone to binges or overeating in general feels powerful cravings to consume; they often see little option but to succumb to these cravings, and this capitulation is a matter of shame and guilt. The language of addiction—“this is a biological drive that I alone cannot control”—fits well with this subjective experience, and it brings a means of communicating distress and helplessness. It may even be helpful in resisting cravings, though whether it is or not is an empirical question that has not been resolved. It is not for the scientist or clinician to police popular language. When a person asserts that they are addicted to sugar, they acknowledge and convey a distressing sense of helplessness and compulsion. The science narrative that is offered that food, like a drug, has affected a person’s brain such that their inhibitory control centers do not work properly, and their reward centers are malfunctioning, is simple and readily accepted. But, the scientist who offers this as the explanation had better be sure that the evidence exists to support it. My view is that it does not. Lacking this evidence, I suggest, severely constrains the explanatory power of the food addiction concept, leaving it perilously close to the sort of “virtus dormitiva” explanation sharply lampooned by Moliere.

## Arguments in favor of food addiction (PJK)

“My drug of choice is food. I use food for the same reasons an addict uses drugs: to comfort, to soothe, to ease stress.”

—Oprah Winfrey

The term “addiction”, even when used in the context of drug abuse, is highly contentious. It means different things to different people; those suffering from the disorder or their loved ones, health-care professionals, researchers, self-help groups, religious organizations, and government agencies all have their own views on what it means to be an addict. For this reason, discrete behavioral characteristics in affected individuals that are considered core features of the disorder have been codified into checklists that are used in the formal diagnosis of substance use disorders (SUDs) [25]. It is little wonder then that the term addiction is so controversial when used in the context of food consumption. I hesitate to position myself as a defender of the concept of food addiction when it is such a nebulous term so open to misinterpretation. However, I consider it self-evident that at least some overweight individuals struggle to control their food intake even when their health and well-being depend on it in a manner that is analogous or even homologous to those affected by SUDs who struggle to control their drug use. After all, SUDs across different classes of drugs with different pharmacological actions are not defined by any shared physiological abnormality that can be objectively measured and used as a diagnostic, such as the elevated blood glucose in the case of diabetes, but rather by the manifestation of a behavioral abnormality that negatively impacts their life: specifically, the failure to control consummatory behavior despite repeated attempts to do so. Consequently, I am a willing defender of the notion that a history of overconsumption of energy-dense palatable food can remodel brain motivation circuits in a manner that renders some overweight individuals persistently vulnerable to the desirable properties of such food, which negatively impacts their health and well-being.

Food addiction, as currently diagnosed using the YFAS and related scales, is considered distinct from obesity. However, the negative consequences associated with a failure to control food intake are most obvious in overweight individuals, who often suffer social stigmatization and are at far greater risk of diabetes, cardiovascular disease, cancer, and other significant health concerns than lean individuals. For this reason, I have framed the arguments below in the context of overweight and obesity, but this should not disguise the fact that many lean individuals also suffer from patterns of dysregulated eating that share many features with SUDs.

Three of the most important clinical features of SUDs are feelings of deprivation when the substance is withheld, a propensity to relapse during periods of abstinence, and consumption that persists despite awareness of negative health, social, financial, or other consequences. Overweight individuals who experience real or perceived social, emotional, or health consequences because of their body weight will often express a desire to lose weight and will repeatedly attempt to do so [26, 27], but limiting their food intake or the types of food that they consume over the prolonged time periods necessary to achieve and maintain a healthy body weight is notoriously difficult. Even those overweight individuals who turn to surgical interventions, highlighting their struggle to exert self-control over their diet, demonstrate remarkably high rates of recidivism, with many gradually increasing their consumption of energy-dense food items, and regaining previously lost body weight over time [28]. Hence, overweight individuals who are unable to exert control over their consummatory behavior, despite awareness of the negative consequences, demonstrate the same core failure to control consumption as those suffering from SUDs.

An issue often raised in the context of food addiction is whether the same brain systems are involved in relapse to weight gain in previously overweight individuals, through their failure to control the amount, frequency, or type of food consumed, as those systems involved in relapse to drug use in SUDs. The underlying assumption being that unless a core brain system involved in drug addiction is similarly impacted in those struggling to control their diet, then the construct of addiction should not be applied to food. There is now a preponderance of human functional imaging data showing that energy-dense palatable food can stimulate changes in the activity of many of the same brain structures and circuits known to be impacted by drugs of abuse. For example, palatable food stimulates reward-relevant activity in the striatum [29–31]. Moreover, weight gain is associated with altered striatal responses to palatable food or cues that predict the availability of such food [32–34], and allelic variation that influences vulnerability to weight gain and obesity can alter reward-relevant activity in the striatum [32, 35]. In addition to the striatum, the activity of other brain areas thought to play an important role in drug addiction, such as prefrontal cortical regions and the amygdala, is similarly altered by consumption of palatable food and development of obesity [36–41]. The animal literature is also replete with evidence that palatable food and drugs of abuse can impact similar brain circuits, particularly the mesoaccumbens dopamine system, and that the development of obesity profoundly alters the function of these same circuits [42–46]. Hence, palatable food and weight gain profoundly impact activity and responsiveness of core components of the brain reward system. But which type of brain abnormality in drug users should be considered the true hallmark of addiction to which changes in the brains of overweight individuals should be compared? It seems unreasonable to assume that there is a specific addiction-relevant pattern of brain activity, at least those patterns that can be detected using current imaging modalities, which can be used to support or refute the existence of food addiction when the very same imaging approaches cannot be used as a diagnostic for drug addiction. Currently, all that can be concluded from these imaging studies is that palatable food and drugs of abuse can impact the function of similar regions of the brain. Ultimately, the value of identifying overlapping brain systems involved in regulating the motivational properties of palatable food and drugs of abuse rests on whether such knowledge facilitates the development of therapeutics that reduce problematic overeating and drug use. Available evidence is promising in this regard. For example, the cannabinoid receptor 1 antagonist rimonabant, developed as an antiobesity drug, facilitates smoking cessation in humans [47]. Similarly, the antiobesity drug lorcaserin, a serotonin 2C receptor antagonist, also facilitates smoking cessation [48]. These findings suggest that common underlying brain processes are likely involved in overeating and drug use.

Another argument often used against the notion of a food-directed use disorder is the question of which ingredient in food is the responsible agent. The underlying assumption here is that only those food items that contain this agent will support addiction-relevant behaviors. Recently, arguments have been made for the involvement of refined sugars [49, 50]. My own view is that it is not necessarily a single macronutrient that is responsible for maladaptive eating but rather the combinations of macronutrients in palatable high-calorie food items that do not occur naturally, but that when combined can pack a supraphysiological punch to brain motivation circuits that is sufficient to modify subsequent consummatory behaviors. Emerging evidence suggests that this may indeed be the case. For example, a recent study in humans found that blended food items high in both fat and carbohydrate were more valued than palatable food items high in fat or carbohydrate alone, and that the blended food had a greater impact on the activity on brain areas involved in reward than the single-nutrient food items [51]. Hence, diets consisting of palatable food items that are rich in energy-dense macronutrients may render less-palatable but healthier food options less attractive and shift dietary preferences toward these highly rewarding, calorie-laden options.

Addiction should not be viewed as a single unitary disorder, related to core deficits in one or more of the same brain system and distinguished only by the users’ drug of choice. Instead, use disorders should be viewed as a constellation of related syndromes that share similar but not entirely overlapping brain and behavior abnormalities, the most conspicuous of which is a failure to control consumption. Indeed, core features of SUDs can differ dramatically depending on which substance is being used, reflecting different underlying neurobiological processes at play. For example, cocaine use disorder is characterized by cycles of binge consumption interspersed by periods of abstinence, with the amounts of cocaine used sufficiently to induce intoxication and often exceeding the amounts that the user wished to limit themselves to, resulting in overt signs of overdosing [52]. By contrast, tobacco use disorder is characterized by remarkably stable and highly regular patterns of daily use, with no overt signs of intoxication, and binge-like consumption not a general feature of the habit [53]. Yet, considering the well-known detrimental health consequences of tobacco smoking, and the struggle that habitual smokers experience when trying to quit, it is difficult to argue that a tobacco smoker is any less “addicted” than someone who binges on cocaine. It is worth pointing out that until recently, tobacco smoking was also the subject of much heated debate about whether it constituted a simple habit or warranted the moniker of full-blown addiction [54, 55]. Before that, the same type of debate centered on cocaine [56]. When viewed from this perspective, it should come as no surprise that overweight individuals who struggle to control their food intake will show brain and behavior abnormalities that are similar in some respects to the prototypic features of psychomotor stimulant, alcohol, tobacco, or opioid addiction, yet differ from these disorders in much the same way that these drug addictions differ from each other. Ultimately, the questions we must ask when considering whether food consumption can, in some circumstances, be considered a use disorder are: does the affected individual fail to exert control over consummation of a substance from which they derive pleasure despite knowledge of potentially severe consequences? Does the affected individual experience feelings of deprivation when the substance is not available or they try to abstain? Does the affected individual show vulnerability to relapse during periods of abstinence? For some overweight individuals at least, the answer to each of these questions is a resounding yes. Precisely, how these features can be codified into checklists of symptoms to identify individuals who are most affected, and the utility of such checklists in a clinical or research setting, should not distract from struggle that some individuals face when trying to exert control over their diet.

Ultimately, I agree with those who express concern about the concept of food addiction. This concept is too nebulous and loaded to convey proper meaning. However, some individuals clearly demonstrate a failure to exert control over their food choices, despite a desire to do so, and as a result experience significant negative consequences. Our ever-increasing understanding of how drugs of abuse remodel motivation circuits in the brain to precipitate compulsive drug use can and should serve as a heuristic to better understand the brain mechanisms of overeating in overweight individuals.

## How to move the field forward

We both agree that questions on the concept of food addiction are heavily confounded by terminology. As noted above, the term addiction is simply too imprecise to be meaningful from a clinical perspective. We also agree that there are patterns of behavior and subjective experiences related to food consumption that bear a resemblance to SUDs, most notably the strong urge to consume, which can become more powerful with abstinence and override personal desires to limit consumption. Where we differ is on the issue of the biological equivalence between those struggling to control their food or drug intake and whether this signifies a deeper resemblance between overeating and drug addiction. More specifically, do strong urges to consume food simply reflect innate biological drives, which can differ between individuals, and which are undoubtedly further influenced by patterns of overconsumption and ensuing weight gain but which should not be conflated with the notion of an addict seeking drugs (Fletcher)? Or, instead, are such urges and associated overeating reflective of a breakdown of behavioral control systems related to neurobiological abnormalities that can be considered analogous or even homologous to those that contribute to SUDs (Kenny)? These differences in opinion speak directly to whether the diagnosis of “food addiction”, as defined by existing instruments such as the YFAS or other instruments that will likely emerge in coming years, captures a legitimate clinical entity that is distinct from other patterns of disordered eating.

A critical evaluation of the concept of food addiction should not seek to undermine the personal and clinical reality of those experiencing strong urges to consume. Instead, the focus should be placed firmly on generating empirical evidence that supports or refutes this concept. Perhaps the most practical scientific strategy to explore the case for food addiction is to begin with our current understanding of how drugs of abuse remodel brain motivation circuits to precipitate compulsive drug seeking, and factors that influence this process. Critically, this approach might adopt two core principles. First, it begins not by assuming that overeating is a form of addiction, although it allows for the possibility that ultimately there may emerge evidence to suggest that at least certain forms of such overconsumption may show high levels of overlap. Rather, the initial aim would be a systematic approach, using insights from the drug addiction literature to focus and sharpen scientific questions relating to obesity. Second, the emphasis would move away from focusing exclusively upon similarities between excessive food and substance use and would acknowledge too the importance of differences in their patterns of use. The functional significance of both the commonalities and the differences in the overuse of food and drugs should be explored together in order to generate a more complete picture of where the food addiction model succeeds and fails. Indeed, as with all useful models, one can learn as much from its limitations as from its success. This is important because we both feel that current characterizations of the food addiction model lack the specificity that would make the model breakable: a sine qua non for a useful model [57]. Overall, we suggest, in demanding less dogmatic or polarized positions, this reframes the question to a much more scientifically compelling and tractable one.

Practical difficulties present themselves when trying to apply drug addiction approaches to the question of food addiction. Even in the case of SUDs, where there is a known agent with an established pharmacology and the picture is not complicated by a preexisting innate biological need for the substance, the brain mechanisms are highly complex, with pronounced variability across individuals and drugs. Despite this complexity, robust molecular, epigenetic, synaptic, cellular, and circuit-based adaptive responses to drugs of abuse, thought to be relevant to the emergence of compulsive drug use in humans, have been detected in laboratory animals [58]. In addition, genetic vulnerabilities that influence these processes have been identified in recent years. Of course, the complexities are magnified significantly in the context of food consumption, which is shaped and regulated by a host of parallel systems with built-in redundancies as well as by complex economic and sociocultural factors. We must consider, therefore, the appreciable challenges when applying concepts in drug addiction to understand food consumption. Nonetheless, some of the important (but by no means the only) concepts from the addiction literature that should be considered in the context of food consumption are explained below.

First, most major drugs of abuse profoundly dysregulate striatal glutamate homeostasis [59], such that environmental stimuli associated with their delivery can evoke “supraphysiological” increases in glutamate spillover, particularly in the nucleus accumbens core [60]. Such cue-evoked increases in glutamate transmission trigger synaptic and structural plasticity in the striatum, reflected by alterations in AMPA/NMDA receptor ratios and new dendritic spine formation [60]. This action, in turn, is thought to contribute to vigorous drug seeking during periods of abstinence. Hence, it will be important to determine whether excessive consumption of palatable foods, and associated weight gain, also induces disruption in striatal glutamate homeostasis, a disruption that may perhaps offer important targets for therapeutic interventions aimed at curbing consumption or preventing relapse. The complexity of applying this aspect of the model to foods should not be underestimated: for example, excessive consumption of palatable foods may not lead to weight gain (e.g., in bulimia nervosa). Moreover, in many cases, particularly of early-onset obesity, primary disturbances lie outside the striatum, for example, within the leptin–melanocortin circuitry of the hypothalamus [61]. Thus, putative striatal alterations will need to be considered within a context of more extensive, albeit subtle, brain changes.

Second, a history of extended access to psychomotor stimulants, alcohol, or opioids in laboratory rodents and in human subjects is associated with profound deficits in brain reward function [62]. It has been hypothesized that drug-induced reward deficits precipitate the emergence of compulsive drug-seeking behaviors [63], and are related to the recruitment of brain aversion and stress systems such as the κ opioid receptor-mediated transmission [64]. Recently, it was shown that overconsumption of palatable energy-dense food and the development of obesity in laboratory rats similarly disrupts brain reward function [65]. While consistent evidence has yet to emerge that this translates to human obesity, this finding suggests that homeostatic adaptive responses occur in brain reward circuits in response to overconsumption of rewarding food or drugs. It will be important to determine if common underlying neuromolecular mechanisms are involved in drug- and food-induced alterations in hedonic responsiveness. Moreover, will amelioration of these adaptive responses in brain reward systems reduce the desire to use food and drugs in humans?

Third, overconsumption of drugs of abuse can facilitate the emergence of habitual-like consummatory behaviors, characterized by their relative insensitivity to the current value of a reinforcer that the animal is responding for [66, 67]. The ability of drugs of abuse to enhance habitual-like patterns of responding may play a role in the persistence of the drug-taking habit. In addition, a history of extended drug access can precipitate drug-taking behaviors that become progressively less sensitive to negative consequences [68, 69]. Emerging evidence suggests that overconsumption of palatable food can similarly precipitate habitual- and compulsive-like patterns of food intake in rats [65, 70] Hence, it will be important to determine if common underlying neurobiological mechanisms contribute to these addiction-relevant patterns of consumption and, again, if manipulating these processes may help to ameliorate overconsumption. It will, however, be profoundly challenging to determine whether rodent findings extend to humans, given the challenges posed by precise but unobtrusive characterization of naturalistic eating behaviors and by difficulties in replicating the conditions under which habit-like responses can be generated, and thereby investigated, in humans (https://osf.io/5pbmz/).

Fourth, drug addiction is often hypothesized to reflect a state of “hypofrontality” in which excessive drug use induces deficits in the function of higher-order cortical centers in the brain, resulting in a progressive loss of executive control over drug-seeking behaviors [71]. Such conceptualizations often consider addiction as a circuit-based disorder, in which top-down signals from the cortex to limbic, basal ganglia, and midbrain regions to inhibit consummatory behavior progressively weaken, whereas bottom-up urges to seek and consume drugs of abuse persist or even strengthen [72]. Recent animal and human findings are consistent with a circuit-based view of drug addiction that is controlled by executive centers in the cortex [71, 73]. Hence, it will be important to thoroughly explore the consequences of overconsumption of palatable energy-dense food on cortical control centers in laboratory animals and humans. Emerging data suggest that higher-order cortical systems indeed undergo robust remodeling in response to such diets [74], but the functional consequences of such cortical remodeling remain poorly understood. Moreover, though ventromedial prefrontal cortex in humans is emerging as a consistent site of structural change in relation to elevated body weight [75], the functional significance of this is by no means clear and functional neuroimaging as a whole does not support any simple notions of hypofrontality as a causative or maintaining factor in obesity.

Fifth, we both agree that using “overeating” as a metric for addiction is problematic, as it is not necessarily the amount of food that is consumed but rather the type and pattern of consumption that is the issue. Drug addiction is often conceptualized as a disorder of decision-making, whereby the value of the drug increases to the point that the user will choose to consume the drug at the expense of competing natural reinforcers or behaviors. In recent years, Ahmed and colleagues have shown that the majority of rodents, when presented with a mutually exclusive choice between a natural reward such as sucrose or a drug reward, such as a cocaine infusion or delivery of alcohol, will select the natural reward over the drug reward [76–80]. Only a small minority of animals will select the drug reward, with this population considered the “addiction vulnerable” animals. Most recently, it was shown that constitutively enhanced GABAergic transmission in the central nucleus of the amygdala may explain the increased value of alcohol in vulnerable animals, as reflected by their choice to consume alcohol at the expense of competing saccharin rewards [81]. Currently, very little is known about the neurobiological mechanisms that regulate the choice to consume palatable energy-dense food at the expense of healthier but less-palatable options and the role that intermittency of access to palatable food can play in influencing such choices [22]. The use of choice procedures similar to those championed by Ahmed to understand maladaptive choice behaviors in the context of drug addiction may facilitate greater understanding of how long-lasting shifts in dietary preferences toward highly rewarding energy-dense food items can occur.

Sixth, large-scale genome-wide association studies (GWAS) in humans are beginning to identify genetic variants that are robustly associated with complex traits or phenotypes, such as cannabis use (https://www.biorxiv.org/content/early/2018/01/08/234294), problematic alcohol use (see https://www.biorxiv.org/content/biorxiv/early/2018/03/08/275917.full.pdf), levels of tobacco smoking [82], and measures of adiposity [83]. If substance use and obesity do share a common etiology, these studies are well suited to identify and characterize it. While early candidate gene studies have suggested such overlap, these have proved to be inconsistent; e.g., ref. [84]. Moreover, initial results from these GWAS suggest that the genetic variants that confer risk for substance use phenotypes do not substantially increase the risk for obesity—if at all. In fact, the results from these GWAS suggest that the common variant genetic architectures of substance use and obesity are largely distinct from one another despite both having high expression levels in brain tissue [83, 85]. It should be borne in mind that this is a field that is developing rapidly, and changing techniques and emerging findings will require careful monitoring with respect to the question at hand.

It is important to bear in mind that while we support the view of drug use as a potential, albeit simplified, model for aberrant food intake, many of the gains that have been made in our understanding of drug use have been based on the reverse modeling process—specifically, by considering it in terms of a hijacking of the systems underlying natural rewards, including foods. There is no contradiction here. While it would certainly be circular to argue, as some have done, that, because foods activate reward circuitry, this suggests that foods are as perilous as drugs, it is nonetheless useful to take an iterative approach in which observations of perturbations associated with substance use can contribute to guiding and interpreting observations related to health-harming overconsumption, and vice versa. But such an approach must acknowledge notable differences between obesity and drug addiction. Reward circuitry, which is the core focus of both, does not function in isolation from other homeostatic and higher-order functions of the brain or from the powerful effects of neural, hormonal, and metabolic signals from the rest of the body. As well as modulating reward-related circuitry [86], these signals have the capacity to shift and shape higher-order cognition and perception [87, 88]. With this in mind, it is important to consider whether mechanisms of appetitive regulation can provide a conceptual framework to better understand drug addiction. All vertebrates have whole-organ systems adapted to sense, consume, digest, and eliminate food, and each level of this process involves exquisitely controlled bidirectional communication between these organ systems and those brain systems involved in appetite control. Consequently, those interested in obesity, binge eating, and other forms of disordered eating do not view the brain in isolation but rather take a holistic view of the body and how it is perturbed in affected individuals. Indeed, slower hormonal and faster vagally transmitted signals from organ systems involved in energy homeostasis, such as the pancreas, liver, and gut, are central to any conceptualization of disordered feeding behaviors and associated diseases. With the exception of alcohol use [89], research conducted into the role of organ systems other than the brain in drug addiction has been sparse. Do peripheral organ systems transmit information to brain motivation circuits in response to drug consumption, and is such peripherally derived information relevant to addiction? In addition to hunger-related signals derived from peripheral organs that act on brain circuits to promote food intake, there are complex systems that serve to inhibit appetite, and failure of such appetite-suppressing systems is often incorporated into conceptualizations of overeating and obesity. Do circuits that inhibit food consumption also play a role in controlling drug intake, and does their dysregulation contribute to the emergence of drug addiction? These are important questions that can help bridge the fields of maladaptive food and drug consumption.

We believe that even those who feel skepticism over the validity and current evidence base of the food addiction model would recognize the potential value and synergy in drawing these fields together. There are many ways in which they may prove mutually informative. But information will be lost if we begin with the assumption that drug addiction processes explain food overconsumption and schedule our empirical endeavors exclusively toward a survey of similarities, some of which are superficial and imprecise. Ultimately, drug addiction could provide a useful model for aspects of food overconsumption, just as consumption of foods and other natural rewards serves a useful purpose to better understand drug addiction. Of course, such synergy may not necessarily translate directly to the clinic [90], but it may well help to guide future targeted therapeutic efforts and contribute to our understanding of whether superficial clinical similarities are underpinned by a deeper resemblance.

## References

[1] Jaspers K (1963). General psychopathology.

[2] Davis C (2014). Evolutionary and neuropsychological perspectives on addictive behaviors and addictive substances: relevance to the “food addiction” construct. Subst Abus Rehabil.

[3] Gearhardt AN, Corbin WR, Brownell KD (2009). Preliminary validation of the Yale Food Addiction Scale. Appetite.

[4] Volkow ND, Wang GJ, Tomasi D, Baler RD (2013). Obesity and addiction: neurobiological overlaps. Obes Rev.

[5] Pedram P, Wadden D, Amini P, Gulliver W, Randell E, Cahill F (2013). Food addiction: its prevalence and significant association with obesity in the general population. PLoS ONE.

[6] Hebebrand J, Albayrak O, Adan R, Antel J, Dieguez C, de Jong J (2014). “Eating addiction”, rather than “food addiction”, better captures addictive-like eating behavior. Neurosci Biobehav Rev.

[7] Schulte EM, Potenza MN, Gearhardt AN (2017). A commentary on the “eating addiction” versus “food addiction” perspectives on addictive-like food consumption. Appetite.

[8] Westwater ML, Fletcher PC, Ziauddeen H (2016). Sugar addiction: the state of the science. Eur J Nutr.

[9] de Vries SK, Meule A (2016). Food addiction and bulimia nervosa: new data based on the Yale Food Addiction Scale 2.0. Eur Eat Disord Rev.

[10] Hilker I, Sanchez I, Steward T, Jimenez-Murcia S, Granero R, Gearhardt AN (2016). Food addiction in bulimia nervosa: clinical correlates and association with response to a brief psychoeducational intervention. Eur Eat Disord Rev.

[11] Meule A, Heckel D, Kubler A (2012). Factor structure and item analysis of the Yale Food Addiction Scale in obese candidates for bariatric surgery. Eur Eat Disord Rev.

[12] Meule A, von Rezori V, Blechert J (2014). Food addiction and bulimia nervosa. Eur Eat Disord Rev.

[13] Gearhardt AN, White MA, Masheb RM, Morgan PT, Crosby RD, Grilo CM (2012). An examination of the food addiction construct in obese patients with binge eating disorder. Int J Eat Disord.

[14] Finlayson G (2017). Food addiction and obesity: unnecessary medicalization of hedonic overeating. Nat Rev Endocrinol.

[15] Long CG, Blundell JE, Finlayson G (2015). A systematic review of the application and correlates of YFAS-diagnosed ‘food addiction’ in humans: are eating-related ‘addictions’ a cause for concern or empty concepts?. Obes Facts.

[16] Wang GJ, Volkow ND, Logan J, Pappas NR, Wong CT, Zhu W (2001). Brain dopamine and obesity. Lancet.

[17] Dang LC, Samanez-Larkin GR, Castrellon JJ, Perkins SF, Cowan RL, Zald DH (2016). Associations between dopamine D2 receptor availability and BMI depend on age. Neuroimage.

[18] Eisenstein SA, Bischoff AN, Gredysa DM, Antenor-Dorsey JA, Koller JM, Al-Lozi A (2015). Emotional eating phenotype is associated with central dopamine D2 receptor binding independent of body mass index. Sci Rep.

[19] Karlsson HK, Tuominen L, Tuulari JJ, Hirvonen J, Parkkola R, Helin S (2015). Obesity is associated with decreased mu-opioid but unaltered dopamine D2 receptor availability in the brain. J Neurosci.

[20] Ziauddeen H, Farooqi IS, Fletcher PC (2012). Obesity and the brain: how convincing is the addiction model?. Nat Rev Neurosci.

[21] Ziauddeen H, Fletcher PC (2013). Is food addiction a valid and useful concept?. Obes Rev.

[22] Corwin RL (2011). The face of uncertainty eats. Curr Drug Abus Rev.

[23] Furlong TM, Jayaweera HK, Balleine BW, Corbit LH (2014). Binge-like consumption of a palatable food accelerates habitual control of behavior and is dependent on activation of the dorsolateral striatum. J Neurosci.

[24] Avena NM, Gearhardt AN, Gold MS, Wang GJ, Potenza MN (2012). Tossing the baby out with the bathwater after a brief rinse? The potential downside of dismissing food addiction based on limited data. Nat Rev Neurosci.

[25] American Psychiatric Association. (2013). Diagnostic and statistical manual of mental disorders.

[26] Puhl RM, Moss-Racusin CA, Schwartz MB, Brownell KD (2008). Weight stigmatization and bias reduction: perspectives of overweight and obese adults. Health Educ Res.

[27] Booth ML, Wilkenfeld RL, Pagnini DL, Booth SL, King LA (2008). Perceptions of adolescents on overweight and obesity: the weight of opinion study. J Paediatr Child Health.

[28] Saunders R (2001). Compulsive eating and gastric bypass surgery: what does hunger have to do with it?. Obes Surg.

[29] Small DM, Zatorre RJ, Dagher A, Evans AC, Jones-Gotman M (2001). Changes in brain activity related to eating chocolate: from pleasure to aversion. Brain.

[30] Stice E, Yokum S, Burger KS, Epstein LH, Small DM (2011). Youth at risk for obesity show greater activation of striatal and somatosensory regions to food. J Neurosci.

[31] Yokum S, Gearhardt AN, Harris JL, Brownell KD, Stice E (2014). Individual differences in striatum activity to food commercials predict weight gain in adolescents. Obesity (Silver Spring).

[32] Stice E, Spoor S, Bohon C, Small DM (2008). Relation between obesity and blunted striatal response to food is moderated by TaqIA A1 allele. Science.

[33] Stice E, Yokum S (2016). Gain in body fat is associated with increased striatal response to palatable food cues, whereas body fat stability is associated with decreased striatal response. J Neurosci.

[34] Stice E, Yokum S, Blum K, Bohon C (2010). Weight gain is associated with reduced striatal response to palatable food. J Neurosci.

[35] van der Klaauw AA, von dem Hagen EA, Keogh JM, Henning E, O’Rahilly S, Lawrence AD (2014). Obesity-associated melanocortin-4 receptor mutations are associated with changes in the brain response to food cues. J Clin Endocrinol Metab.

[36] Holsen LM, Zarcone JR, Thompson TI, Brooks WM, Anderson MF, Ahluwalia JS (2005). Neural mechanisms underlying food motivation in children and adolescents. Neuroimage.

[37] Killgore WD, Weber M, Schwab ZJ, Kipman M, DelDonno SR, Webb CA (2013). Cortico-limbic responsiveness to high-calorie food images predicts weight status among women. Int J Obes (Lond).

[38] Killgore WD, Young AD, Femia LA, Bogorodzki P, Rogowska J, Yurgelun-Todd DA (2003). Cortical and limbic activation during viewing of high- versus low-calorie foods. Neuroimage.

[39] Scholtz S, Miras AD, Chhina N, Prechtl CG, Sleeth ML, Daud NM (2014). Obese patients after gastric bypass surgery have lower brain-hedonic responses to food than after gastric banding. Gut.

[40] Siep N, Roefs A, Roebroeck A, Havermans R, Bonte ML, Jansen A (2009). Hunger is the best spice: an fMRI study of the effects of attention, hunger and calorie content on food reward processing in the amygdala and orbitofrontal cortex. Behav Brain Res.

[41] van Bloemendaal LRGIJ, Ten Kulve JS, Barkhof F, Konrad RJ, Drent ML (2014). GLP-1 receptor activation modulates appetite- and reward-related brain areas in humans. Diabetes.

[42] Geiger BM, Haburcak M, Avena NM, Moyer MC, Hoebel BG, Pothos EN (2009). Deficits of mesolimbic dopamine neurotransmission in rat dietary obesity. Neuroscience.

[43] Martel P, Fantino M (1996). Influence of the amount of food ingested on mesolimbic dopaminergic system activity: a microdialysis study. Pharmacol Biochem Behav.

[44] Bassareo V, Di Chiara G (1999). Modulation of feeding-induced activation of mesolimbic dopamine transmission by appetitive stimuli and its relation to motivational state. Eur J Neurosci.

[45] Blackburn JR, Phillips AG, Jakubovic A, Fibiger HC (1986). Increased dopamine metabolism in the nucleus accumbens and striatum following consumption of a nutritive meal but not a palatable non-nutritive saccharin solution. Pharmacol Biochem Behav.

[46] Wilson C, Nomikos GG, Collu M, Fibiger HC (1995). Dopaminergic correlates of motivated behavior: importance of drive. J Neurosci.

[47] Cahill K, Ussher M. Cannabinoid type 1 receptor antagonists (rimonabant) for smoking cessation. Cochrane Database Syst Rev: 2007;CD005353. 10.1002/14651858.CD005353.pub3 17943852

[48] Shanahan WR, Rose JE, Glicklich A, Stubbe S, Sanchez-Kam M (2017). Lorcaserin for smoking cessation and associated weight gain: a randomized 12-week clinical trial. Nicotine Tob Res.

[49] Ahmed SH, Guillem K, Vandaele Y (2013). Sugar addiction: pushing the drug-sugar analogy to the limit. Curr Opin Clin Nutr Metab Care.

[50] Colantuoni C, Rada P, McCarthy J, Patten C, Avena NM, Chadeayne A (2002). Evidence that intermittent, excessive sugar intake causes endogenous opioid dependence. Obes Res.

[51] DiFeliceantonio AG, Coppin G, Rigoux L, Thanarajah SE, Dagher A, Tittgemeyer M (2018). Supra-additive effects of combining fat and carbohydrate on food reward. Cell Metab.

[52] Decorte T (2001). Quality control by cocaine users: underdeveloped harm reduction strategies. Eur Addict Res.

[53] National Center for Chronic Disease Prevention and Health Promotion (US) Office on Smoking and Health. Patterns of tobacco use among U.S. youth, young adults, and adults. In: (US)CfDCaP, editor. The health consequences of smoking-50 years of progress: a report of the surgeon general. US Department of Health and Human Services, Atlanta, GA; 2014.

[54] Russell MA (1974). The smoking habit and its classification. Practitioner.

[55] Warburton DM (1989). Is nicotine use an addiction?. Psychologist.

[56] Gawin FH (1991). Cocaine addiction: psychology and neurophysiology. Science.

[57] Teufel C, Fletcher PC (2016). The promises and pitfalls of applying computational models to neurological and psychiatric disorders. Brain.

[58] Russo SJ, Dietz DM, Dumitriu D, Morrison JH, Malenka RC, Nestler EJ (2010). The addicted synapse: mechanisms of synaptic and structural plasticity in nucleus accumbens. Trends Neurosci.

[59] Kalivas PW (2004). Glutamate systems in cocaine addiction. Curr Opin Pharmacol.

[60] Scofield MD, Heinsbroek JA, Gipson CD, Kupchik YM, Spencer S, Smith AC (2016). The nucleus accumbens: mechanisms of addiction across drug classes reflect the importance of glutamate homeostasis. Pharmacol Rev.

[61] O’Rahilly S, Farooqi IS (2008). Human obesity: a heritable neurobehavioral disorder that is highly sensitive to environmental conditions. Diabetes.

[62] Kenny PJ, Hoyer D, Koob GF (2018). Animal models of addiction and neuropsychiatric disorders and their role in drug discovery: honoring the legacy of Athina Markou. Biol Psychiatry.

[63] Koob GF, Le Moal M (2008). Addiction and the brain antireward system. Annu Rev Psychol.

[64] Wee S, Koob GF (2010). The role of the dynorphin-kappa opioid system in the reinforcing effects of drugs of abuse. Psychopharmacology (Berl).

[65] Johnson PM, Kenny PJ (2010). Dopamine D2 receptors in addiction-like reward dysfunction and compulsive eating in obese rats. Nat Neurosci.

[66] Ersche KD, Gillan CM, Jones PS, Williams GB, Ward LH, Luijten M (2016). Carrots and sticks fail to change behavior in cocaine addiction. Science.

[67] Everitt BJ, Robbins TW (2005). Neural systems of reinforcement for drug addiction: from actions to habits to compulsion. Nat Neurosci.

[68] Deroche-Gamonet V, Belin D, Piazza PV (2004). Evidence for addiction-like behavior in the rat. Science.

[69] Vanderschuren LJ, Everitt BJ (2004). Drug seeking becomes compulsive after prolonged cocaine self-administration. Science.

[70] Tantot F, Parkes SL, Marchand AR, Boitard C, Naneix F, Laye S (2017). The effect of high-fat diet consumption on appetitive instrumental behavior in rats. Appetite.

[71] Chen BT, Yau HJ, Hatch C, Kusumoto-Yoshida I, Cho SL, Hopf FW (2013). Rescuing cocaine-induced prefrontal cortex hypoactivity prevents compulsive cocaine seeking. Nature.

[72] Diana M, Raij T, Melis M, Nummenmaa A, Leggio L, Bonci A (2017). Rehabilitating the addicted brain with transcranial magnetic stimulation. Nat Rev Neurosci.

[73] Terraneo A, Leggio L, Saladini M, Ermani M, Bonci A, Gallimberti L (2016). Transcranial magnetic stimulation of dorsolateral prefrontal cortex reduces cocaine use: a pilot study. Eur Neuropsychopharmacol.

[74] Thompson JL, Drysdale M, Baimel C, Kaur M, MacGowan T, Pitman KA (2017). Obesity-induced structural and neuronal plasticity in the lateral orbitofrontal cortex. Neuropsychopharmacology.

[75] Medic N, Ziauddeen H, Ersche KD, Farooqi IS, Bullmore ET, Nathan PJ (2016). Increased body mass index is associated with specific regional alterations in brain structure. Int J Obes (Lond).

[76] Freese L, Durand A, Guillem K, Ahmed SH. Pre-trial cocaine biases choice toward cocaine through suppression of the nondrug option. Pharmacol Biochem Behav. 2018;173:65–73. 10.1016/j.pbb.2018.07.010 30056175

[77] Huynh C, Fam J, Ahmed SH, Clemens KJ (2017). Rats quit nicotine for a sweet reward following an extensive history of nicotine use. Addict Biol.

[78] Lenoir M, Cantin L, Vanhille N, Serre F, Ahmed SH (2013). Extended heroin access increases heroin choices over a potent nondrug alternative. Neuropsychopharmacology.

[79] Madsen HB, Ahmed SH (2015). Drug versus sweet reward: greater attraction to and preference for sweet versus drug cues. Addict Biol.

[80] Cantin L, Lenoir M, Augier E, Vanhille N, Dubreucq S, Serre F (2010). Cocaine is low on the value ladder of rats: possible evidence for resilience to addiction. PLoS ONE.

[81] Augier E, Barbier E, Dulman RS, Licheri V, Augier G, Domi E (2018). A molecular mechanism for choosing alcohol over an alternative reward. Science.

[82] Thorgeirsson TE, Geller F, Sulem P, Rafnar T, Wiste A, Magnusson KP (2008). A variant associated with nicotine dependence, lung cancer and peripheral arterial disease. Nature.

[83] Locke AE, Kahali B, Berndt SI, Justice AE, Pers TH, Day FR (2015). Genetic studies of body mass index yield new insights for obesity biology. Nature.

[84] Munafo MR, Matheson IJ, Flint J (2007). Association of the DRD2 gene Taq1A polymorphism and alcoholism: a meta-analysis of case-control studies and evidence of publication bias. Mol Psychiatry.

[85] Sanchez-Roige S, Gray JC, MacKillop J, Chen CH, Palmer AA (2018). The genetics of human personality. Genes Brain Behav.

[86] Farooqi IS, Bullmore E, Keogh J, Gillard J, O’Rahilly S, Fletcher PC (2007). Leptin regulates striatal regions and human eating behavior. Science.

[87] Owens AP, Allen M, Ondobaka S, Friston KJ (2018). Interoceptive inference: from computational neuroscience to clinic. Neurosci Biobehav Rev.

[88] Tallon-Baudry C, Campana F, Park HD, Babo-Rebelo M (2018). The neural monitoring of visceral inputs, rather than attention, accounts for first-person perspective in conscious vision. Cortex.

[89] Morris LS, Voon V, Leggio L. Stress, motivation, and the gut-brain axis: a focus on the ghrelin system and alcohol use disorder. Alcohol Clin Exp Res. 2018. 10.1111/acer.13781 PMC6252147 29797564

[90] Wilson GT (2010). Eating disorders, obesity and addiction. Eur Eat Disord Rev.

