# Correction: Food addiction: a valid concept?

**DOI:** 10.1038/s41386-018-0288-1

**Published:** 2018-12-07

**Authors:** Paul C. Fletcher, Paul J. Kenny

**Affiliations:** 1 Department of Psychiatry, University of Cambridge, Cambridge, CB2 8AH UK; 2 Cambridgeshire and Peterborough NHS Foundation Trust, Cambrdge, CB21 5EF UK; 3 Department of Neuroscience, Icahn School of Medicine at Mount Sinai, New York, NY 10029 USA

Correction to: *Neuropsychopharmacology*; 10.1038/s41386-018-0203-9

This article was originally published under standard licence, but has now been made available under a [CC BY 4.0] license. The PDF and HTML versions of the paper have been modified accordingly.

